# From a free electron gas to confined states: A mixed island of PTCDA and copper phthalocyanine on Ag(111)

**DOI:** 10.3762/bjnano.13.131

**Published:** 2022-12-22

**Authors:** Alfred J Weymouth, Emily Roche, Franz J Giessibl

**Affiliations:** 1 Institute of Experimental and Applied Physics, Department of Physics, University of Regensburg, 93053 Regensburg, Germany

**Keywords:** AFM, copper phthalocyanine, d*I*/d*V*, PTCDA, STM

## Abstract

When perylene-3,4,9,10-tetracarboxylic dianhydride (PTCDA) is deposited on the Ag(111) surface at submonolayer coverage, it forms islands under which the native Shockley state of the Ag(111) surface can no longer be found. Previous work has shown that this state shifts upwards to form a new interface state starting at 0.6 V above the Fermi level, having properties of a two-dimensional electron gas (2DEG). We investigated mixed islands of PTCDA and copper phthalocyanine (CuPc) to study the change in the electronic state with the addition of an electron donor. We no longer observe a 2DEG state and instead identify states at 0.46 and 0.79 V. While one state appears in d*I*/d*V* images as an array of one-dimensional quantum wells, our analysis shows that this state does not act as a free electron gas and that the features are instead localized above individual PTCDA molecules.

## Introduction

Organic semiconductor devices typically include a metal–organic interface. At this interface, it is important to be able to modify the band structure to optimize the efficiency of a device [1]. One of the most successful methods to change the electronic structure of a molecular semiconductor device is to add a second molecular species either at low concentration as a dopant or at higher concentrations as a mixed layer [2].

Perylenetetracarboxylic dianhydride (PTCDA) is an organic molecule that has been investigated for its properties as an organic semiconductor and as a dye. It is straightforward to evaporate in vacuum and, at submonolayer coverage, lies flat on metal surfaces. Submonolayer coverage of PTCDA on Ag(111) is known to form islands with a herringbone reconstruction [3–4]. These islands are hosts to an interface state that acts like a free-electron gas [5]. This interface state has been observed with two-photon photoelectron spectroscopy experiments [6–8], and has been studied with density functional theory (DFT) [9–10]. Previous work [11] has used d*I*/d*V* spectroscopy as a measurement of the density of electronic states [12] and identified this interface state starting at 0.6 eV. One characteristic of a two-dimensional electron gas (2DEG) is that standing waves can be observed near defects as a result of scattering [13]. Sabitova et al. [11] acquired d*I*/d*V* images at various voltages above 0.6 V and observed wave-like patterns around the defects. We reproduced these measurements as can be seen in Figure S1, Supporting Information File 1.

A natural complement to PTCDA is copper phthalocyanine (CuPc) for several reasons: First, CuPc and PTCDA together can form an organic light-emitting diode with PTCDA as the acceptor [14]. Second, CuPc [15] and PTCDA both lie flat on metal surfaces at submonolayer coverage, enabling high-precision STM and atomic force microscopy (AFM) scanning. Third, CuPc and PTCDA are known to form commensurate phases on flat metal surfaces. In particular, they have been well studied at different stoichiometries on Ag(111) [16]. Henneke and co-workers showed that more than 0.15 ML of PTCDA in addition to 0.5 ± 0.1 ML of CuPc are required to form mixed islands of CuPc and PTCDA [16]. Within these conditions, there can be different stoichiometries within the mixed islands, including a phase with a 1:1 ratio of PTCDA to CuPc within the unit cell, called the PC phase, and a phase with a 2:1 ratio of PTCDA to CuPc within each unit cell, called the P_2_C phase [16]. A STM and AFM investigation of single CuPc and PTCDA molecules on a thin insulating layer interestingly showed little change of the d*I*/d*V* spectra (features shifted, but were preserved) or of the corresponding d*I*/d*V* images when the two molecules were close to each other implying little direct interaction [17]. Stadtmüller et al. extensively studied the P_2_C phase with STM, d*I*/d*V* measurements, and DFT calculations [18]. They showed that while an isolated CuPc molecule on Ag(111) has a level that is half-filled, this level shifts above the Fermi level when the CuPc is embedded in a P_2_C island, indicating that CuPc donates charge to PTCDA [18]. While this investigation of the mixed phase concentrated on electronic states below the Fermi level, electronic states above the Fermi level have been studied with CuPc and PTCDA on Ag(111) in a stacked configuration [19]. When CuPc is on top of PTCDA, the interface state can still be observed [19], and CuPc has a strong bond to the underlying PTCDA layer [20].

In this paper, we present STM and AFM data of P_2_C and PC phases on Ag(111), concentrating on the PC phase. The high-resolution AFM allows us to precisely image the molecular configuration of the mixed phase. In contrast to previous studies that focussed on understanding the electronic states below the Fermi level [18], we focus on the electronic states above the Fermi level. Our d*I*/d*V* measurements show a loss of the free electron-like behaviour as seen in the PTCDA/Ag(111) interface state. We identify a state that appears to be an array of one-dimensional quantum wells based on its shape in the d*I*/d*V* spatial maps. However, we do not observe scattering at defects and conclude that it is localized laterally at the PTCDA molecules.

## Methods

Measurements were carried out in a He-bath scanning probe microscope (CreaTec Fischer & Co. GmbH) and were acquired in ultrahigh vacuum at 5.6 K. Ag(111) (Mateck GmbH) was prepared with standard sputter and anneal cycles. The PTCDA and CuPc were evaporated from a custom-built evaporator. A detailed description of the sample preparation is available in Supporting Information File 1. A qPlus AFM/STM sensor [21] with an etched W-tip was used. Tunneling spectoscopy data (d*I*/d*V* data) were acquired with a lock-in amplifier included in the control electronics (Nanonis from SPECS GmbH). The AC signal had a frequency of 879 Hz, and we used a modulation voltage amplitude of 20 mV after ensuring that spectra did not change in shape with modulation voltages between 5 and 20 mV. The bias voltage and AC signal were applied to the sample. AFM data were acquired in frequency-modulation mode [22] with a sensor oscillation amplitude of 50 pm. The resonance frequency of the sensor is 38819 Hz, which is much higher than the modulation voltage used for spectroscopy data.

## Results and Discussion

**Figure 1:**
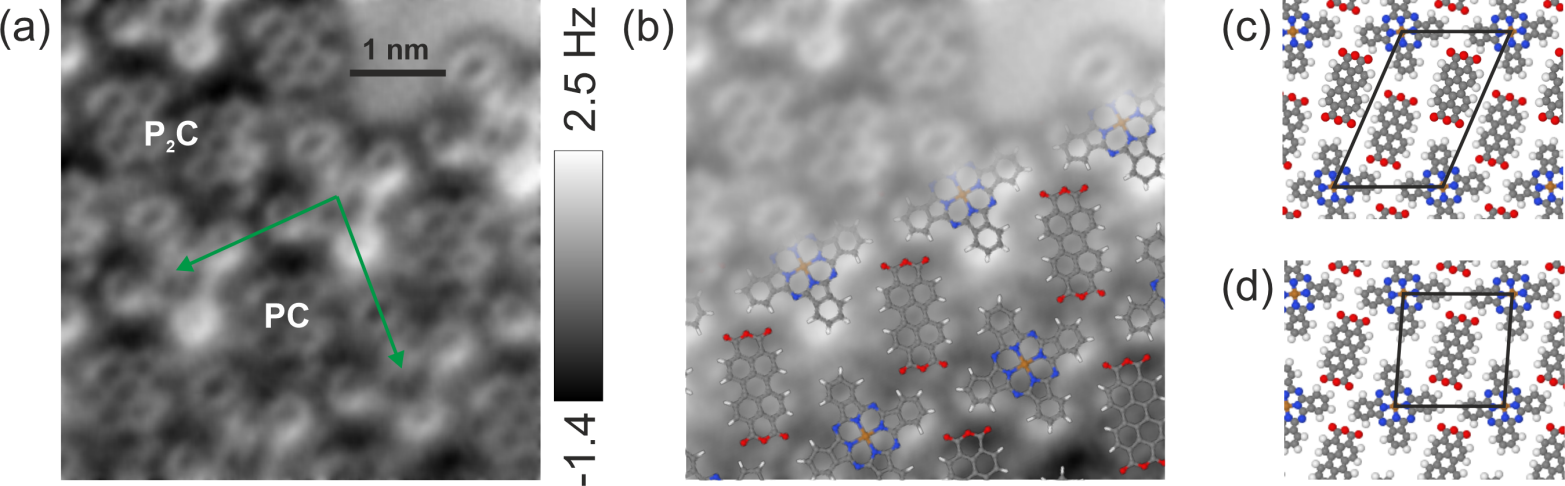
(a) Constant-height AFM image (plane-subtracted) above an area with two local stoichiometries: with two PTCDA molecules for each CuPc (P_2_C) and with a one-to-one mixture (PC). The unit cell of the PC phase is indicated by the green arrows. (b) The same image with ball-and-stick figures to guide the eye. (c) Ball-and-stick model of the P_2_C phase with the unit cell shown in black. (d) Ball-and-stick model of the PC phase with the unit cell shown in black.

Figure 1a is an AFM image of an island showing both PC and P_2_C phases. The internal structure of the molecules appears similar to images taken with a CO-terminated tip [23]. However, it could be due to another molecule at the tip apex leading to similar contrast as has been previously discussed in the literature [24]. As there was a slight drift in the vertical direction, the AFM data was plane-subtracted to enhance the contrast. The raw data is given in Figure S2 of Supporting Information File 1. In the lower half of the image, an area labelled PC can be seen. The unit cell of the PC phase is indicated by the green arrows in Figure 1a and includes one PTCDA molecule and one CuPc molecule. An area of a double-row of PTCDA molecules can be seen in the upper left side of the image, labelled P_2_C. In the P_2_C phase, there is an extra PTCDA molecule in the unit cell. The P_2_C and PC phases are shown schematically in Figure 1c,d. These two stoichiometries were previously presented, and this AFM image confirms the structures proposed from SPA-LEED and STM experiments [25]. As reported previously [16], the PTCDA molecules within a given phase all have the same orientation.

The lower lobes of the CuPc molecules appear brighter, which might be an indication that the CuPc molecules do not lie flat in this configuration. This would be different from recent experimental work that showed that individual CuPc molecules adsorb on Ag(111) in a planar configuration [26]. However, it might also be an artefact of an asymmetric tip. If the CuPc molecules lie above the surface at a greater height than the PTCDA molecules, then the effect of an asymmetric tip would be more pronounced above them. The contrast in the AFM images indicate that the CuPc molecules are higher, and previous experimental evidence has shown that CuPc sits higher on the surface than PTCDA if they are not in a mixed phase [18]. We are not aware of experimental data regarding their heights in the mixed PC phase.

**Figure 2:**
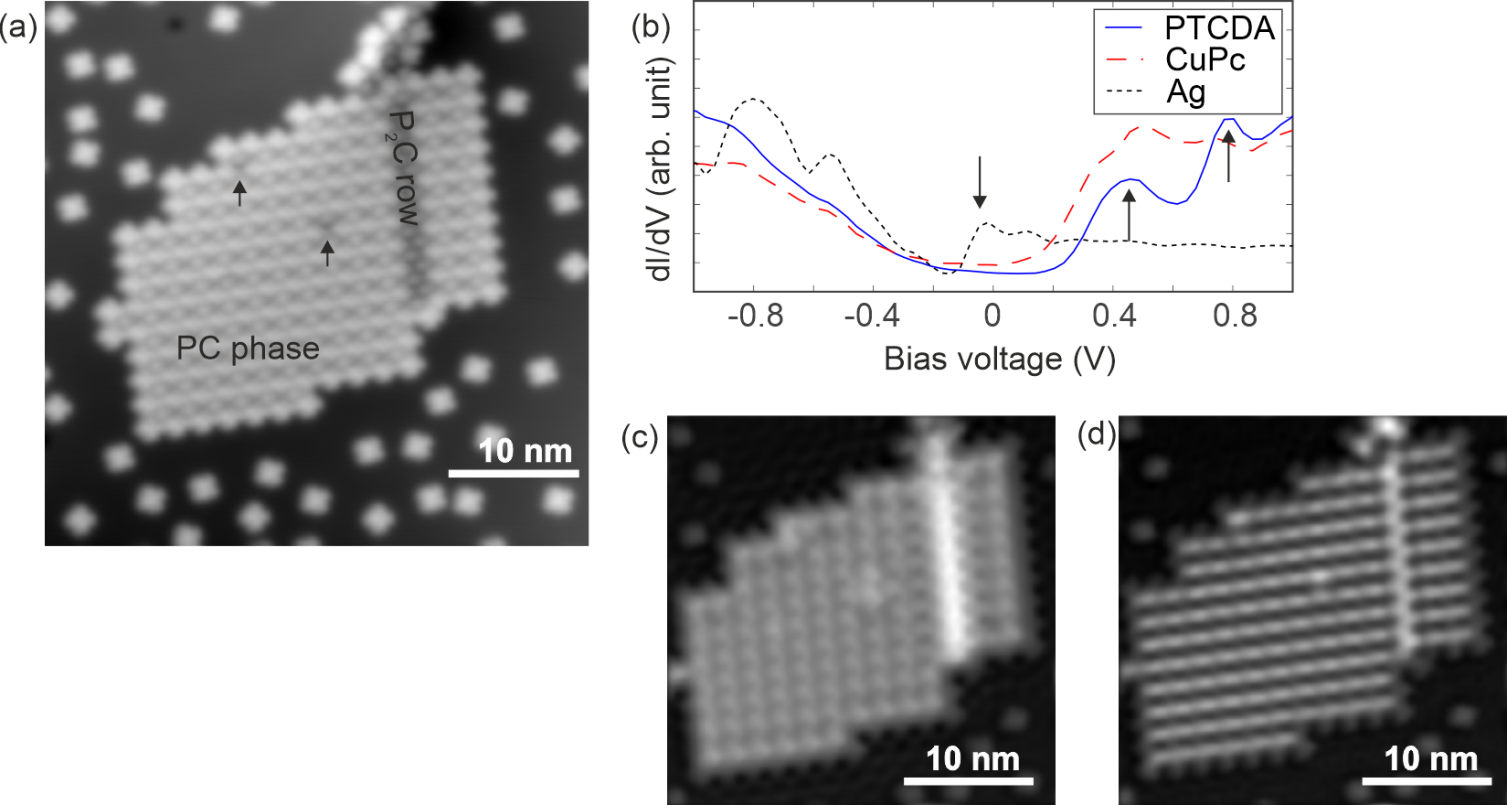
(a) STM image of a mixed-phase island surrounded by individual CuPc molecules. (b) d*I*/d*V* spectrum above two molecules in the PC phase (PTCDA and CuPc), as well as over the bare Ag(111) surface. The arrows show the relevant features of two states above the PTCDA molecules and the onset of the surface state of the bare Ag(111) surface. (c) d*I*/d*V* image taken at 0.5 V. (d) d*I*/d*V* image taken at 0.78 V.

In Figure 2a, a STM image of an island of PTCDA and CuPc is shown. Most of the island consists of the PC phase, and there is a row of P_2_C indicated in the figure. The growth conditions required for these mixed islands result in surfaces with islands of the PC phase, often with a single row of P_2_C, as well as large islands of pure PTCDA and lone CuPc adsorbates around the islands. While the small amount of P_2_C is not relevant to this study, we found it an unavoidable byproduct of our preparation. It is not clear why we observe the P_2_C phase as isolated rows within the PC islands. A further dataset showing both a pure PC island and a large PTCDA island is shown in Figure S3 and Figure S4 of Supporting Information File 1. At two positions on the island shown in Figure 2a, there are unknown defects that are indicated by black arrows.

We acquired d*I*/d*V* spectra above a PTCDA molecule in the PC island, a CuPc molecule in the PC island, and near the island on the bare Ag(111) surface. The spatial locations of the spectra above the molecules are shown in Figure S5 of Supporting Information File 1. In the d*I*/d*V* data shown in Figure 2b, the expected surface state above the bare Ag(111) surface can be seen below the Fermi level, indicated by the downward-pointing arrow. The spectrum has drastically changed above the PTCDA molecules that are involved in the PC phase of the island, shown as a solid blue line in Figure 2b (compared to, e.g., [5] or Figure S1c in Supporting Information File 1). Two features can be seen, as indicated by the upward-pointing arrows.

As discussed in the Introduction, [18] showed that CuPc on Ag(111) without PTCDA has a spectral peak around the Fermi level (the F-LUMO peak), whereas when CuPc is in a P_2_C island, the F-LUMO peak has emptied and is above the Fermi level. Their DFT calculations show the F-LUMO peak to be around 0.2 V, and the d*I*/d*V* spectra show features at 0.6 V [18]. Figure 2b does not contain a peak in the d*I*/d*V* spectra over the CuPc molecules at the Fermi level but rather a peak between the Fermi level and 1 V. This indicates that also in the PC phase, the energy level straddling the Fermi level has shifted to higher energies and that, in the PC island, CuPc acts as a donor.

**Figure 3:**
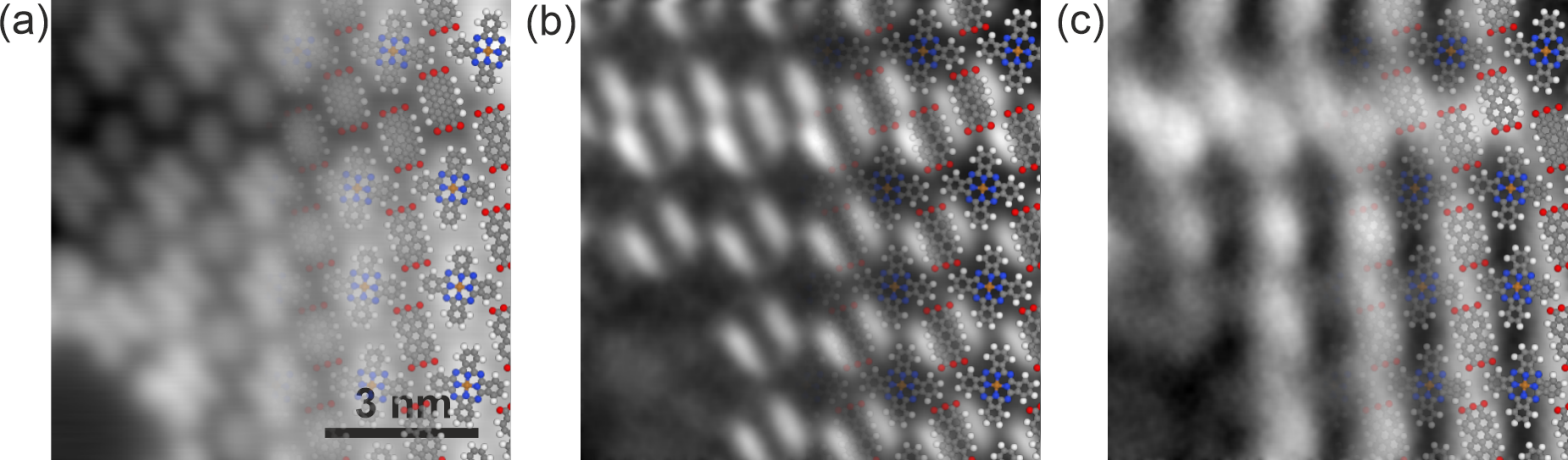
(a) STM image of a PC island with a row of P_2_C; 0.36 V, 100 pA. (b) d*I*/d*V* image at −0.39 V, showing two lobes at each PTCDA molecule. This can be used to orient the ball-and-stick figures that are overlain on each subfigure. (c) d*I*/d*V* image at 0.79 V showing that this state, responsible for the stripe pattern, is localized above the PTCDA molecules.

To investigate the spatial dependence of the spectral features indicated in Figure 2b, we acquired d*I*/d*V* images at 0.5 V and at 0.78 V, as shown in Figure 2c,d. Figure 2c shows a square pattern over the island that is enhanced at the location of the P_2_C row. Interestingly, Figure 2d shows stripes that run through the PC phase. As we will show with Figure 3, the stripe pattern in Figure 2d is localized above PTCDA molecules.

In Figure 2d, the stripes appear to connect neighbouring molecules. If the state were spatially delocalized over all molecules of a single stripe, as the interface state of a pure PTCDA island is delocalized over all PTCDA molecules of the island, we would expect it to act as a free-electron state confined to one dimension. However, neither Figure 2c nor Figure 2d show indications of the standing wave features near defects that are observed for a pure PTCDA island (shown in [11] and Figure S1b in Supporting Information File 1). We therefore conclude that this state we observe in Figure 3d is indeed a state localized at single PTCDA molecules.

To further investigate the nature of the stripe pattern shown in Figure 2d, we collected data at higher resolution. Figure 3 shows images of a different island that also includes both PC and P_2_C phases. In Figure 3a, the STM image clearly shows the CuPc and PTCDA molecules. The orientation of the PTCDA molecules can be verified by acquiring d*I*/d*V* spectra at −0.39 V, which is an energy level corresponding to the localized LUMO level [27], which we refer to as the F-LUMO. The F-LUMO state (although not obviously present in Figure 2b as a local maximum in the spectrum) can be clearly seen in Figure 3b where two lobes correspond to a single PTCDA molecule [5]. It is noteworthy that the F-LUMO spectral feature that is present in pure PTCDA on Ag islands can also be observed on the PC island, whereas the states above the Fermi level have dramatically changed. This is most likely because the filled states of PTCDA are not as affected by the presence of an electron donor (CuPc) as the states at or above the Fermi level are. The state responsible for the stripe pattern is shown in Figure 3c and is spatially localized above the PTCDA molecules.

At this point, it is tempting to consider the possibility of locally “tuning” the strength of the interface state by doping a PTCDA island with a diminishing amount of CuPc molecules and observing the interface state around them. However, at lower concentrations, CuPc does not integrate into PTCDA islands, but rather only decorates their borders. Therefore a gradual tuning is not possible; adding CuPc at a great enough concentration only presents the ability to form a proper mixed layer where the interface state is no longer present.

Instead of doping with CuPc, another possibility to tune the interface state would be to investigate a mixed phase with a phthalocyanine molecule that exhibits different electrical properties, such as has been reported for a mixed phase of tin phthalocyanine (SnPc) with PTCDA [28]. SnPc acts very similarly to CuPc, except the aforementioned F-LUMO is not completely depleted but remains partially filled [28].

## Conclusion

Both PTCDA and CuPc are archetypical molecules used to forward our understanding of acceptor–donor pairs on surfaces. In this letter, we presented an investigation of the PC phase with AFM, STM, d*I*/d*V* spectra, and d*I*/d*V* imaging, concentrating on the states above the Fermi level. From the CuPc spectrum (Figure 2b), we propose that, similar to its behaviour in P_2_C islands [18], CuPc acts as a donor in PC islands. This donation leaves states localized at the PTCDA under the Fermi level relatively unchanged, but drastically changes the unfilled states above the Fermi level. We showed the existence of a spectral feature near 0.4 V, which appears spatially as a square pattern, and a spectral feature near 0.8 V, which appears as a stripe pattern. Neither spectral feature shows evidence of the free-electron gas behaviour that is seen in the interface state in islands of pure PTCDA.

## Supporting Information

The Supporting Information includes methods, scattering in a PTCDA island, raw data for Figure 2, additional d*I*/d*V* data, and locations of spectra taken in Figure 3.

File 1 Additional experimental data.

## References

[1] Lüssem B, Keum C-M, Kasemann D, Naab B, Bao Z, Leo K (2016). Chem Rev.

[2] Walzer K, Maennig B, Pfeiffer M, Leo K (2007). Chem Rev.

[3] Umbach E, Glöckler K, Sokolowski M (1998). Surf Sci.

[4] Eremtchenko M, Schaefer J A, Tautz F S (2003). Nature.

[5] Temirov R, Soubatch S, Luican A, Tautz F S (2006). Nature.

[6] Schwalb C H, Sachs S, Marks M, Schöll A, Reinert F, Umbach E, Höfer U (2008). Phys Rev Lett.

[7] Schwalb C H, Marks M, Sachs S, Schöll A, Reinert F, Umbach E, Höfer U (2010). Eur Phys J B.

[8] Marks M, Armbrust N, Güdde J, Höfer U (2020). New J Phys.

[9] Rohlfing M, Temirov R, Tautz F S (2007). Phys Rev B.

[10] Dyer M S, Persson M (2010). New J Phys.

[11] Sabitova A, Temirov R, Tautz F S (2018). Phys Rev B.

[12] Lang N D (1986). Phys Rev B.

[13] Crommie M F, Lutz C P, Eigler D M (1993). Nature.

[14] Cao G-H, Qin D-S, Guan M, Cao J-S, Zeng Y-P, Li J-M (2007). Chin Phys Lett.

[15] Grand J-Y, Kunstmann T, Hoffmann D, Haas A, Dietsche M, Seifritz J, Möller R (1996). Surf Sci.

[16] Henneke C, Felter J, Schwarz D, Stefan Tautz F, Kumpf C (2017). Nat Mater.

[17] Cochrane K A, Roussy T S, Yuan B, Tom G, Mårsell E, Burke S A (2018). J Phys Chem C.

[18] Stadtmüller B, Lüftner D, Willenbockel M, Reinisch E M, Sueyoshi T, Koller G, Soubatch S, Ramsey M G, Puschnig P, Tautz F S (2014). Nat Commun.

[19] Lerch A, Zimmermann J E, Namgalies A, Stallberg K, Höfer U (2018). J Phys: Condens Matter.

[20] Stadtmüller B, Sueyoshi T, Kichin G, Kröger I, Soubatch S, Temirov R, Tautz F S, Kumpf C (2012). Phys Rev Lett.

[21] Giessibl F J (1998). Appl Phys Lett.

[22] Albrecht T R, Grütter P, Horne D, Rugar D (1991). J Appl Phys.

[23] Gross L, Mohn F, Moll N, Liljeroth P, Meyer G (2009). Science.

[24] Sweetman A M, Jarvis S P, Sang H, Lekkas I, Rahe P, Wang Y, Wang J, Champness N R, Kantorovich L, Moriarty P (2014). Nat Commun.

[25] Stadtmüller B, Henneke C, Soubatch S, Tautz F S, Kumpf C (2015). New J Phys.

[26] Weymouth A J, Riegel E, Simmet B, Gretz O, Giessibl F J (2021). ACS Nano.

[27] Kraft A, Temirov R, Henze S K M, Soubatch S, Rohlfing M, Tautz F S (2006). Phys Rev B.

[28] van Straaten G, Franke M, Soubatch S, Stadtmüller B, Duncan D A, Lee T-L, Tautz F S, Kumpf C (2018). J Phys Chem C.

