# Supplementary material for: From a free electron gas to confined states: A mixed island of PTCDA and copper phthalocyanine on Ag(111)
Source: Beilstein J Nanotechnol. 2022 Dec 22;13:1572–7. doi: 10.3762/bjnano.13.131 (PMC9795859; doi:10.3762/bjnano.13.131)
Supplement: File 1 — Additional experimental data. [file Beilstein_J_Nanotechnol-13-1572-s001.pdf]

BEILSTEIN JOURNAL OF NANOTECHNOLOGY

Supporting Information

for

From a free electron gas to confined states: A mixed island of PTCDA and copper phthalocyanine on Ag(111)

Alfred J. Weymouth, Emily Roche and Franz J. Giessibl

Beilstein J. Nanotechnol. **2022,** *13,* 1572–1577. doi:10.3762/bjnano.13.131

Additional experimental data

License and Terms: This is a supporting information file under the terms of the Creative Commons Attribution License (https://creativecommons.org/licenses/by/4.0). Please note that the reuse, redistribution and reproduction in particular requires that the author(s) and source are credited and that individual graphics may be subject to special legal provisions.
The license is subject to the *Beilstein Journal of Nanotechnology* terms and conditions: (https://www.beilstein-journals.org/bjnano/terms)

Methods

A single-crystal Ag(111) sample was cleaned by repeated cycles of sputtering and annealing (650 °C) until it was clean as verified by STM and AFM images.

PTCDA and CuPc were deposited from a custom-built evaporator. Molecules were always deposited on a surface that is nominally at room temperature. The molecules are held in a small quartz crucible that is heated by tungsten wire wrapped around the crucible. The evaporator has a shutter on such that deposition times can be precisely controlled. It does not have thermocouples, and therefore we cannot report the temperature during deposition, only the current through the wire. Deposition rates were too small to be accurately determined by the quartz microbalance on the opposite side of the chamber, and therefore the evaporation rate needed to be determined by STM images.

A mixed phase with a PC island, as reported by Henneke et al [15], requires approximately 0.5 ML coverage of CuPc (where 1 ML corresponds to complete coverage of the bare metal with molecules adsorbed flat on the surface) and more than 0.15 ML of PTCDA. The islands are then formed, surrounded by individual CuPc molecules, which repel each other. As a comment to STM/AFM experimentalists, this means that there is very little bare metal surface for tip preparation.

During one preparation, first PTCDA was deposited (warm-up of 6 min at a current of 1.3 A and deposited for 20 s at 1.25 A). Then CuPc was deposited (warm-up of 9 min at 1.51 A, 1 min at 1.71 A and then deposited at 1.71 A for 2 min). Upon investigating the surface, we concluded that more PTCDA was required and deposited again with the sample at room temperature (warm up 6 min at 1.3 A, deposit at 1.25 A for 20 s). We then observed mixed phases that were not reported previously in literature. In order to form the expected PC and P_2C phases, we annealed the sample to 100 °C.

A second preparation started again with clean Ag. Then CuPc (warm-up 9 min at 1.51 A, 1 min at 1.71 A and then deposit at 1.71 A for 30 s) and PTCDA (warm-up 6 min at 1.3 A, 1 min at 1.25 A then deposit 1.25 A for 30 s) were deposited. The sample was then annealed. This resulted in too few CuPc molecules, and so stepwise CuPc was further deposited (three times, each warming up 9 min at 1.51 A, then warming at 1.71 A for 1 min, then depositing at 1.71 A for 1 min, then annealing at 100 °C for 10 min) until the coverage of CuPc was adequate to form the PC phases.

Scattering in a PTCDA island

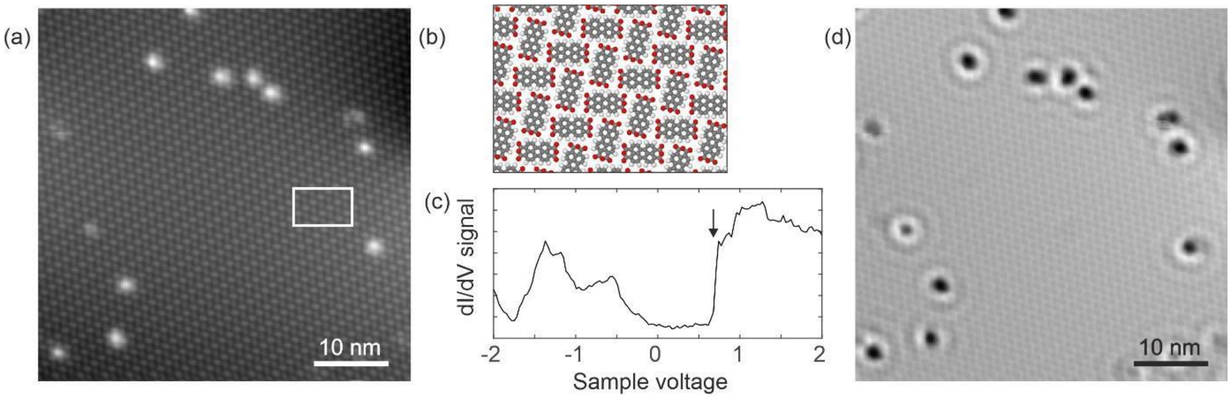

Figure S1: (a) STM image of a large PTCDA island. (0.7 V, 200 pA) (b) The ball-and-stick figure shows a model of the indicated area in (a). (c) dI/dV spectra taken over a PTCDA molecule, showing the onset of the interface state by the black arrow at 0.6 V. (d) dI/dV image at 0.85 V shows a standing wave pattern around unknown defects in the PTCDA island.

Raw data for Figure 2

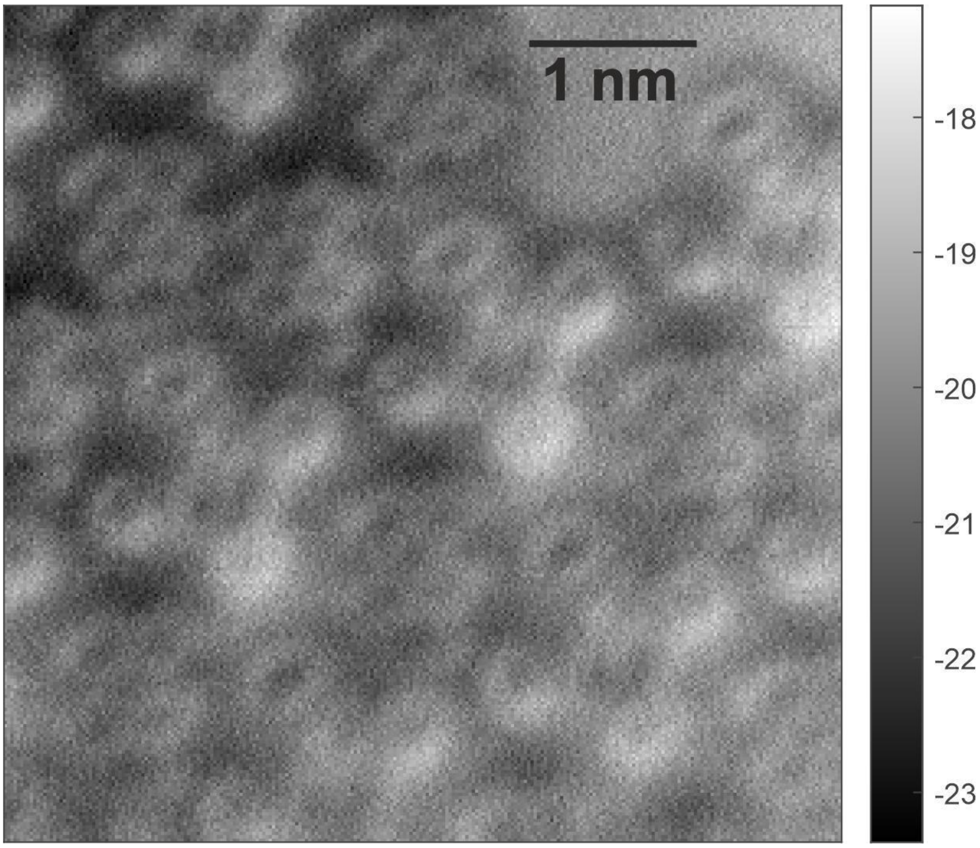

Figure S2: Raw AFM data (given in Hz) of the data shown in Figure 2. The overall planar contrast change is due to a small amount of creep during image acquisition. Therefore, the plane-subtracted AFM image is shown in the main text.

Additional dI/dV data

We acquired several datasets above various islands. In one dataset, we observed a pure PC island, free CuPc molecules and a PTCDA island that was bordered by CuPc molecules. At a bias voltage of 0.4 V, the square pattern can again be seen on the PC island, and at 0.8 V the stripe pattern can also be seen. dI/dV images were taken of the same area (30 nm × 30 nm) and are presented in Figure S4.

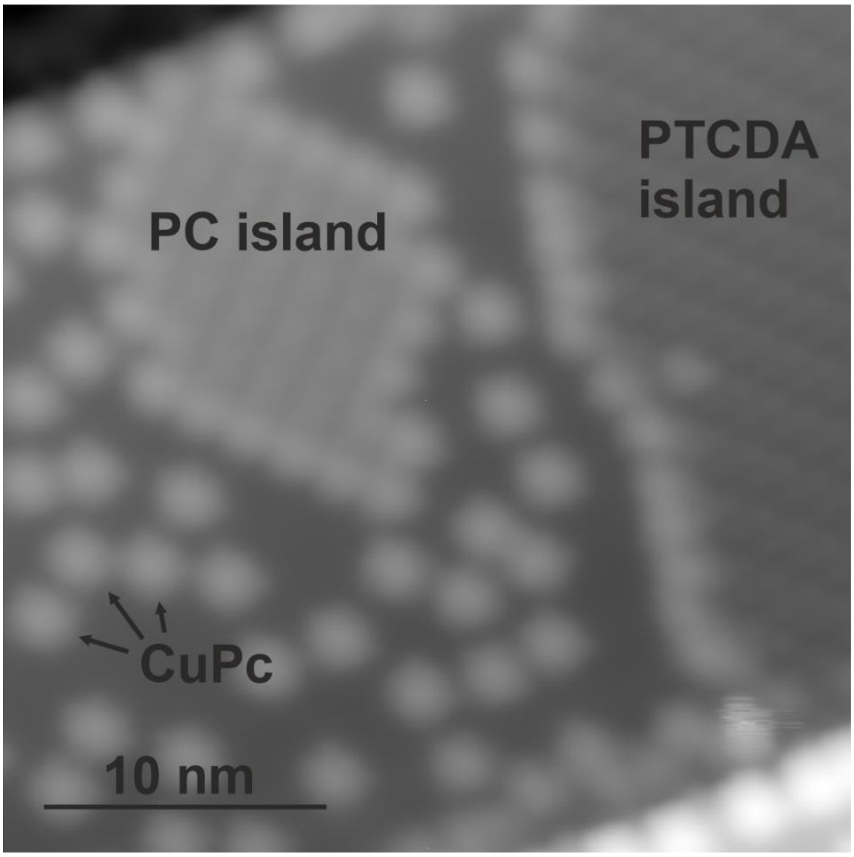

Figure S3: STM topography at 0.3 V. The PC island and the PTCDA island are identified. Several CuPc adsorbates are explicitly indicated.

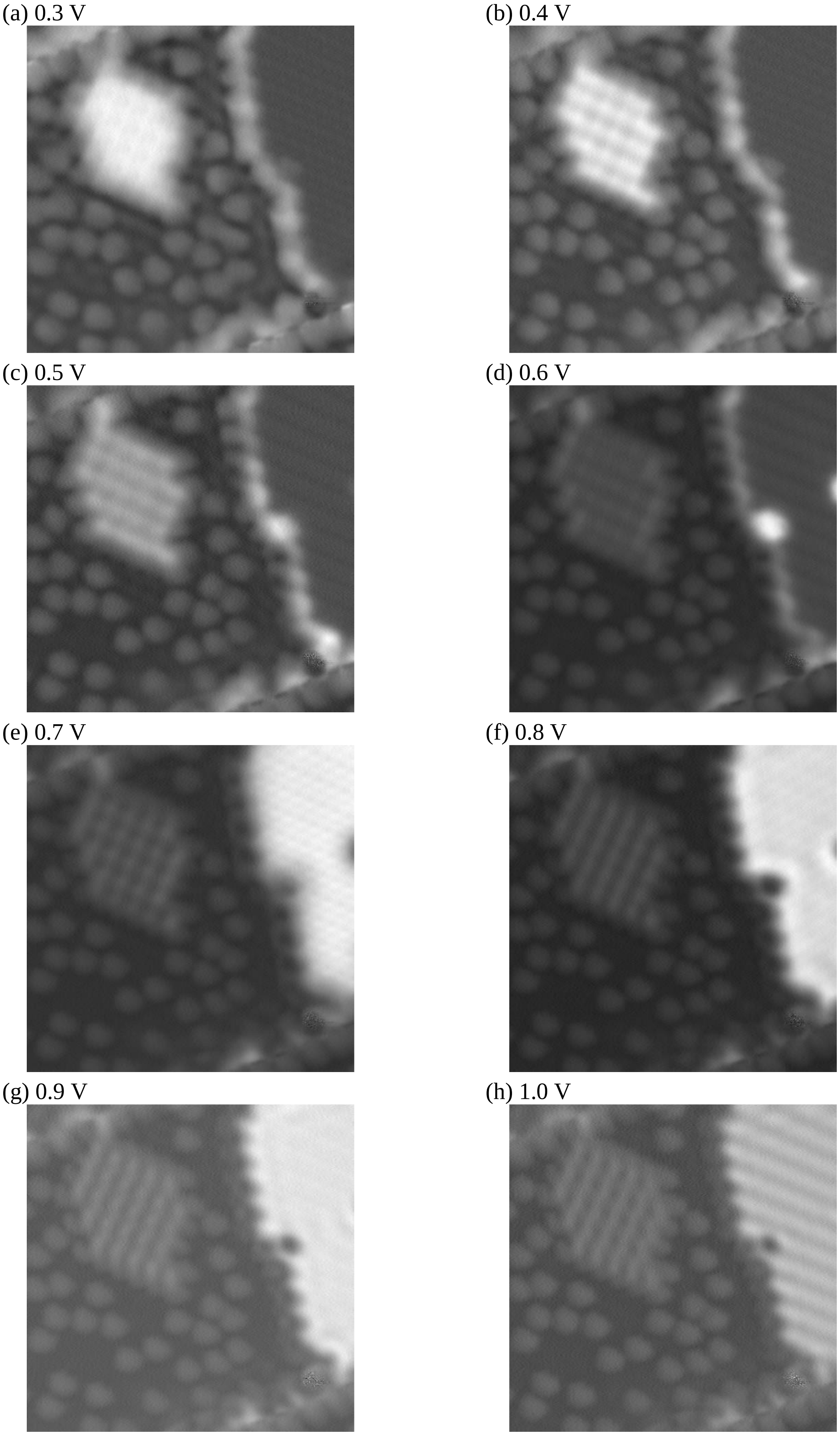

Figure S4: d*I*/d*V* images of the same area as Figure S2 at voltages listed.

Locations of spectra taken in Figure 3

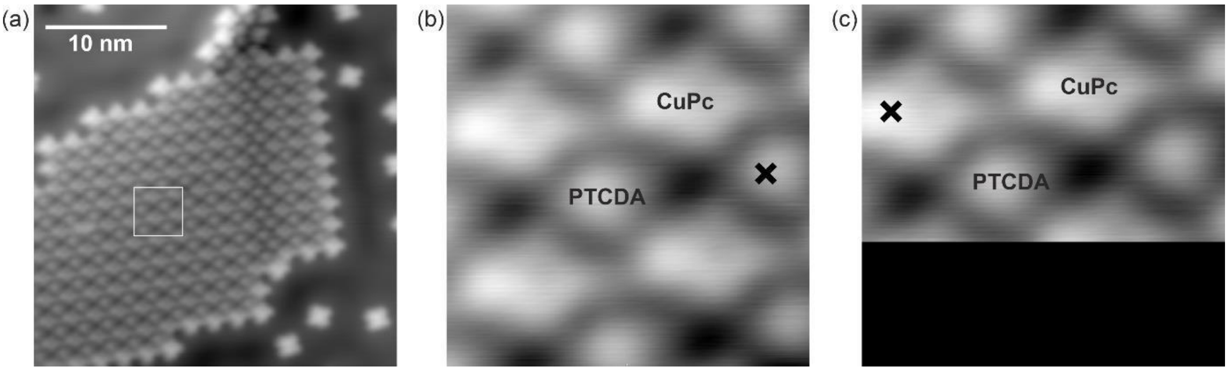

Figure S5: Spectral locations marked with “×” of the data in Figure 3b. (a) Once the island was located, a region of interest within the island was determined. STM topography at 0.1 V, 100 pA. (b) STM image within the island used to determine the species and the location for the spectrum over PTCDA. (c) STM image (halted partway through, thus the black underside) used to determine the location for the spectrum over CuPc.
